# Supplementary material for: Differential Protein Expression in Extracellular Vesicles Defines Treatment Responders and Non-Responders in Multiple Sclerosis
Source: Int J Mol Sci. 2024 Oct 6;25(19):10761. doi: 10.3390/ijms251910761 (PMC11477160; doi:10.3390/ijms251910761)
Supplement: Supplementary file 1 [file ijms-25-10761-s001.zip › ijms-3232424-supplementary.pdf]

Supplemental table 1: Differentially expressed proteins between responders and non-responders in T cells; B cells; neurons and oligodendrocytes-derived extracellular vesicles.

Cell	R/NR	Name	p-value	Fold Change
T cells	Responder PRE	2B14	0,05	0,13
		MMP8	0,04	0,20
		F13A	0,01	0,22
		CAH1	0,01	0,31
		PON3	0,00	0,35
		ETFB	0,02	0,36
		LV151	0,02	0,38
		RS2	0,04	0,40
		CYB5	0,02	0,41
		1433T	0,03	0,43
		M2OM	0,02	0,43
		ERP29	0,04	0,46
		HBAT	0,02	0,47
	Non-responder PRE	MBL2	0,03	2,12
		ENOG	0,01	2,14
		DAF	0,01	2,15
		RS10	0,04	2,22
		HNRPM	0,04	2,25
		TMED9	0,03	2,37
		NPM	0,04	2,52
		LAMB2	0,01	2,72
		RBM14	0,01	3,00
		SFXN3	0,02	3,58
		EFHD2	0,01	3,77
		HORN	0,05	3,90
		FN3K	0,01	3,91
		SAA2	0,01	8,27
		PSB5	0,00	9,12

T cells	Non-responder POST	BCAM	0,00	0,09
		LV39	0,02	0,16
		PIMT	0,01	0,23
		UFL1	0,02	0,26
		A1AT	0,02	0,36
		FCN2	0,05	0,44
		6PGD	0,03	0,44
		PTN6	0,01	0,46
		ERP29	0,01	0,47
		433B	0,04	0,48
		THRB	0,00	0,49
	Non-responder PRE	PIGR	0,02	2,05
		ACON	0,04	2,21
		RS10	0,02	2,54
		HS105	0,02	2,56
		MYO1C	0,00	2,67
		TCPH	0,01	3,03
		SAP	0,02	3,05
		HNRPM	0,00	3,13
		CKAP4	0,01	3,19
		PGM2	0,01	3,42
		CD151	0,03	3,56
		PLOD2	0,01	3,70
		TMED9	0,01	3,75
		EFHD2	0,01	4,32
		TAF2	0,03	4,57
		BDH2	0,03	4,83
		ABHEB	0,01	5,19
		XPO2	0,01	5,22
		TADBP	0,02	5,35
		PSB5	0,01	5,50
		ETFA	0,02	5,62
		SAA2	0,01	5,97
		GP1BA	0,04	10,37

T cells	Responder PRE	CLIC4	0,00	9,90
		ARP3	0,00	6,20
		RETN	0,02	3,94
		CN37	0,05	3,66
		CYB5	0,01	3,59
		RL31	0,00	3,58
		SND1	0,00	3,33
		CD151	0,01	3,11
		LFA3	0,02	3,02
		HEXB	0,03	2,95
		UTRO	0,04	2,71
		SPTN1	0,05	2,67
		PON3	0,04	2,59
		CY24B	0,03	2,58
		ITA6	0,01	2,40
		CD44	0,03	2,32
		LV151	0,04	2,22
		ILEU	0,01	2,20
		HBAT	0,02	2,16
		HNRPU	0,00	2,03
	Responder POST	SFXN3	0,03	0,46
		NACAM	0,04	0,45
		KIN17	0,03	0,43
		LMNA	0,00	0,40
		MYG	0,04	0,34
		RS14	0,04	0,30
		SAA2	0,05	0,27
		CRIP2	0,04	0,26
		CMC1	0,04	0,24
		NAGK	0,01	0,08

T cells	Non-responder POST	CN37	0,04	5,84
		PSA2	0,02	3,01
		1433B	0,02	2,86
		SCRB2	0,03	2,74
		CALU	0,02	2,74
		ANXA5	0,00	2,73
		PDIA4	0,02	2,61
		ANXA3	0,01	2,59
		SAP	0,03	2,38
		PECA1	0,04	2,37
		RD23A	0,04	2,20
		SERC5	0,05	2,20
		PERL	0,04	2,19
		SDHA	0,02	2,18
		PERM	0,04	2,04
	Responder POST	CRP	0,00	0,36
		R4RL2	0,01	0,35
		MYG	0,03	0,35
		CUL4B	0,05	0,33
		PSB4	0,04	0,28
		PLOD2	0,02	0,22
		XPO2	0,00	0,17
		IQGA1	0,02	0,14

B cells	Responder PRE	RAP1B	0,03	0,07
		CAH2	0,00	0,17
		HS90A	0,00	0,25
		COL11	0,02	0,28
		TAGL	0,03	0,28
		RASK	0,04	0,30
		APOC3	0,02	0,34
		EF2	0,01	0,36
		PGRC2	0,03	0,36
		DMBT1	0,02	0,43
		CLIC1	0,04	0,46
		XDH	0,02	0,49
	Non-responder PRE	SF3B1	0,02	2,02
		CYB5	0,01	2,16
		CKAP4	0,05	2,43
		TOP1	0,04	2,63
		ILEU	0,02	2,81
		TALDO	0,01	3,86
		DNJC5	0,04	4,27
		GDIR2	0,01	4,39
		PSMD7	0,00	5,74
		GNAQ	0,00	12,40

B cells	Non-responder PRE	SEP11	0,02	2,04
		SC23A	0,04	2,08
		4F2	0,00	2,12
		PGBM	0,01	2,15
		SCRB1	0,03	2,16
		ECP	0,03	2,17
		CO9	0,01	2,31
		CNRP1	0,02	2,41
		CRIP2	0,03	2,56
		LRP1	0,01	2,57
		KV113	0,04	2,74
		KCRM	0,01	3,53
		LV310	0,01	3,81
		CPT1A	0,01	3,86
		TALDO	0,02	3,91
		RHAG	0,04	4,02
	Non-responder POST	SPTN1	0,02	0,15
		PSB5	0,00	0,16
		PERL	0,02	0,23
		FA9	0,01	0,29
		PSB1	0,02	0,30
		PGH1	0,03	0,31
		LV39	0,00	0,33
		AOFB	0,00	0,33
		RIR1	0,01	0,35
		LANC1	0,05	0,35
		ADDB	0,02	0,36
		QOR	0,02	0,39
		SFXN3	0,04	0,40
		CAVN1	0,05	0,40

B cells	Responder POST	AMY1	0,01	0,50
		LDHA	0,02	0,47
		LYAG	0,01	0,40
		LYAM1	0,01	0,36
		RS20	0,02	0,33
		TYPH	0,04	0,29
		HNRH3	0,01	0,26
		PSMD6	0,02	0,25
		LAMC1	0,04	0,24
		GDIR2	0,01	0,15
		DNJC5	0,05	0,10
	Responder PRE	RAP1B	0,03	8,85
		GRAN	0,00	6,14
		COL11	0,01	5,27
		RD23A	0,00	4,70
		MYO1C	0,05	3,81
		PRS10	0,01	3,35
		KCRB	0,05	2,89
		DSG1	0,04	2,71
		ERO1A	0,02	2,55
		CD151	0,03	2,43
		ROA1	0,05	2,31
		H2AY	0,05	2,22
		H10	0,04	2,14
		HNRL1	0,03	2,09
		CAH2	0,02	2,04

B cells	Responder POST	PSMD6	0,01	0,15
		ADA10	0,00	0,16
		KCRM	0,03	0,18
		LRP1	0,01	0,22
		RL24	0,04	0,23
		ANXA2	0,02	0,28
		HNRH3	0,01	0,29
		PTPRC	0.00	0,29
		EMIL1	0,03	0,42
		CRP	0,02	0,44
		ALDOA	0,04	0,46
		C1QB	0,02	0,46
		SODM	0,03	0,48
	Non-responder POST	VNN1	0,04	2,07
		HV102	0,01	2,28
		TINAL	0,02	2,31
		RS15A	0,03	2,37
		PIGR	0,00	2,48
		HNRL1	0,03	2,52
		SAA2	0,03	3,22
		LV39	0,00	3,65
		PRS10	0,02	3,88
		VDAC2	0,02	5,12

Neuron	Non-responder PRE	PDC6I	0,02	2,04
		BAP31	0,04	2,13
		NID1	0,03	2,25
		DNJA2	0,05	2,39
		S14L3	0,01	2,44
		CATC	0,05	2,45
		MYL6	0,02	2,49
		HV343	0,00	2,52
		RL7	0,02	3,94
		ACO13	0,00	3,99
	Responder PRE	VDAC2	0,03	0,17
		HSPB6	0,01	0,18
		PSB5	0,01	0,26
		GLYM	0,05	0,29
		EF1G	0,01	0,32
		COX2	0,02	0,35
		RS23	0,02	0,36
		ANGT	0,05	0,36
		RS19	0,00	0,37

Neuron	Non-responder PRE	MCM7	0,00	10,32
		GGT1	0,00	10,22
		COTL1	0,02	5,93
		K1C14	0,00	3,72
		RL7	0,03	3,44
		AL5AP	0,05	3,31
		APOC3	0,02	3,29
		SAMP	0,02	3,27
		APOA	0,00	3,09
		PGH1	0,01	2,95
		PERL	0,02	2,76
		PDC6I	0,02	2,58
		LIMC1	0,01	2,40
		AMPB	0,03	2,29
		HV118	0,04	2,29
		ZPI	0,00	2,13
		ITIH4	0,00	2,11
		EDIL3	0,00	2,09
	Non-responder POST	G6PI	0,03	0,39
		FETUB	0,04	0,34
		SERA	0,00	0,29
		IVD	0,02	0,26
		CMC1	0,02	0,07
		LDLR	0,03	0,02

Neuron	Responder PRE	ECHA	0,01	5,02
		CLIC4	0,02	4,09
		QCR1	0,03	3,92
		MT1X	0,01	3,15
		CD14	0,05	2,96
		CY24B	0,03	2,90
		SPEE	0,04	2,88
		COPA	0,03	2,64
		PRTN3	0,05	2,40
		CCAR2	0,05	2,36
		MFGM	0,05	2,26
		PDIA4	0,02	2,17
		MGA	0,01	2,14
		FHR4	0,05	2,10
		TCPH	0,04	2,03
	Responder POST	LAMA5	0,02	0,49
		LV545	0,04	0,50
		SPNT	0,03	0,45
		URP2	0,04	0,44
		PLTP	0,00	0,37
		NCAM	0,00	0,37
		PSB8	0,05	0,37
		TTC38	0,05	0,36
		BPI	0,01	0,36
		PYC	0,05	0,24
		GNAQ	0,01	0,22
		TAGL2	0,00	0,15

Neuron	Responder POST	TTC38	0,01	0,11
		KAP2	0,05	0,19
		PLTP	0,00	0,20
		GUAA	0,03	0,23
		LEG1	0,03	0,27
		DERM	0,02	0,41
		ZPI	0,01	0,42
		PCYOX	0,04	0,43
		NONO	0,02	0,48
		CISY	0,03	0,49
	Non-responder POST	SERA	0,01	2,12
		SAMH1	0,04	2,14
		DEF4	0,03	2,19
		GNAI2	0,03	2,39
		DMBT1	0,02	2,45
		TCPQ	0,01	2,49
		LIS1	0,02	2,55
		RB11B	0,01	2,64
		APMAP	0,01	2,80
		ECHA	0,02	5,33

Oligodendrocyte	Responder PRE	EST1	0,01	0,50
		A1AG2	0,00	0,49
		CD47	0,00	0,46
		B3AT	0,04	0,46
		DAF	0,03	0,46
		RACK1	0,02	0,46
		PGBM	0,04	0,46
		KV133	0,02	0,45
		BPIB2	0,02	0,45
		CYC	0,03	0,44
		IC1	0,02	0,44
		CRK	0,02	0,42
		ROA1	0,00	0,40
		PSA2	0,04	0,40
		ACACA	0,04	0,40
		DEMA	0,05	0,36
		FBLN3	0,03	0,35
		LV140	0,03	0,34
		IGHG2	0,01	0,29
		AATM	0,03	0,28
		CDC42	0,00	0,20
		VASP	0,02	0,18
		KV106	0,04	0,18
		RS11	0,00	0,09
		DDX21	0,01	0,07
	Non-responder PRE	THAS	0,00	9,41
		DHPR	0,04	4,76
		MYO1C	0,03	4,43
		SYIC	0,00	3,93
		VNN1	0,03	3,50
		HNRPC	0,01	3,15
		COPB2	0,02	2,72
		TMED9	0,04	2,70
		FCL	0,05	2,69
		CO6A1	0,02	2,65
		PGRC1	0,00	2,65
		HV349	0,01	2,63
		GCN1	0,03	2,53
		HEMO	0,01	2,41
		CO9	0,03	2,22
		GPX1	0,03	2,21
		THRB	0,01	2,06
		CAH2	0,00	2,05

		A2AP	0,01	2,02

Oligodendrocyte	Non-responder PRE	RNAS2	0,01	8,79
		ECHB	0,01	5,52
		MYO1C	0,03	4,94
		COPA	0,02	4,83
		ARC1B	0,01	4,39
		SYIC	0,02	3,90
		TADBP	0,02	3,67
		KV113	0,02	3,56
		ADDB	0,04	2,93
		ARP3	0,05	2,86
		HV349	0,01	2,46
		ZA2G	0,04	2,06
		LIPL	0,02	2,01
	Non-responder POST	ZYX	0,04	0,48
		PTN6	0,03	0,47
		TYPH	0,04	0,44
		PRS10	0,04	0,44
		DPP4	0,02	0,44
		NID2	0,02	0,44
		CCD25	0,01	0,43
		APOA	0,03	0,42
		EST1	0,03	0,40
		SC23A	0,05	0,39
		SRSF6	0,02	0,38
		RL23	0,03	0,36
		RS20	0,03	0,36
		HBA	0,02	0,35
		CD47	0,01	0,34
		CAMP	0,03	0,32
		RTN4	0,05	0,29

Oligodendrocyte	Responder PRE	HV349	0,01	0,49
		MATN2	0,03	0,48
		RINI	0,02	0,45
		A2AP	0,01	0,45
		PDIA4	0,03	0,44
		PRDX1	0,00	0,41
		CO6A1	0,01	0,39
		GLPC	0,02	0,40
		RAC2	0,04	0,38
		HEMO	0,00	0,38
		CAZA1	0,00	0,37
		TPM1	0,02	0,34
		CHLE	0,05	0,34
		SNTB2	0,04	0,34
		ACON	0,02	0,33
		LBP	0,02	0,33
		CAPG	0,04	0,31
		LASP1	0,04	0,27
		PRAX	0,05	0,23
		BCAM	0,01	0,22
		ALBU	0,01	0,21
		COPB2	0,00	0,21
		XPO2	0,01	0,19
		THAS	0,00	0,19
		EDIL3	0,02	0,16
		PTPRC	0,01	0,16
	Responder POST	CYC	0,00	5,55
		COEA1	0,00	4,71
		PLTP	0,02	3,65
		RS26	0,01	3,44
		CHIT1	0,03	3,20
		KIN17	0,02	3,03
		KV311	0,01	3,02
		F13B	0,05	2,75
		NP1L4	0,02	2,50
		EM55	0,05	2,49
		ROA1	0,01	2,45
		IGKC	0,03	2,29
		CO3	0,00	2,22
		IMA1	0,02	2,16
		TCPQ	0,01	2,06
		SERPH	0,03	2,01

Oligodendrocyte	Non-responder POST	CNRP1	0,03	2,00
		NGAL	0,02	2,01
		RLA0	0,02	2,08
		PRDX6	0,01	2,11
		ELNE	0,04	2,17
		AQP1	0,02	2,32
		MARE1	0,04	2,37
		TCPQ	0,02	2,41
		LEG3	0,02	2,45
		TTC38	0,04	2,50
		KV401	0,04	2,63
		PRKDC	0,05	2,93
		HPTR	0,02	3,23
		A1AT	0,02	3,58
		AGO02	0,00	4,58
	Responder POST	LASP1	0,01	0,07
		PSB5	0,03	0,11
		PTPRC	0,01	0,13
		BAP31	0,05	0,18
		XPO2	0,01	0,21
		GBB1	0,04	0,25
		CD59	0,00	0,25
		ARC1B	0,03	0,26
		TOM34	0,02	0,30
		GDIR2	0,03	0,31
		PLST	0,05	0,32
		PSMD6	0,04	0,33
		PSMD3	0,00	0,34
		MYH11	0,00	0,35
		LBP	0,02	0,35
		PLIN3	0,04	0,37
		KV113	0,01	0,38
		MYPT1	0,01	0,39
		COPB2	0,01	0,40
		ERAP1	0,03	0,40
		CALX	0,02	0,41
		RAC2	0,01	0,43
		FA5	0,02	0,46
		CD9	0,02	0,46
		CTND1	0,00	0,46
		PDIA4	0,04	0,47
		RALA	0,02	0,49
		STRAP	0,04	0,49
